# Supplementary material for: Body Mass Index-Specific Nanoparticle Protein Corona Signatures in Late Pregnancy
Source: bioRxiv. 2026 May 27:2026.05.23.727413. Preprint. [Version 1] doi: 10.64898/2026.05.23.727413 (PMC13232142; doi:10.64898/2026.05.23.727413)
Supplement: Supplement 1 [file media-1.docx]

**Body Mass Index-Specific Nanoparticle Protein Corona Signatures in Late Pregnancy**

Samantha Velazquez^1^, Matthew C. Juber^2^, Michele Okun^3*^, Edward Lau^2*^, Ali Akbar Ashkarran^4,5*^

^1^Department of Biology, University of Colorado Colorado Springs, Colorado Springs, CO, USA

^2^Department of Medicine, University of Colorado School of Medicine, Aurora, CO, USA

^3^Sleep and Biobehavioral Health Research Laboratory, BioFrontiers Center, University of Colorado Colorado Springs, Colorado Springs, CO, USA

^4^Department of Physics and Energy Science, University of Colorado Colorado Springs, Colorado Springs, CO, USA

^5^BioFrontiers Center, University of Colorado Colorado Springs, Colorado Springs, CO, USA

***Corresponding authors:** (MO, EL, AAA) emails: ([mokun@uccs.edu](mailto:mokun@uccs.edu); [edward.lau@cuanschutz.edu](mailto:edward.lau@cuanschutz.edu); [aashkarr@uccs.edu](mailto:aashkarr@uccs.edu))

**Figure S1:** TEM images of PC-coated NPs following incubation with NW (a-c), OW (d-f), and OB (g-i) third-trimester pregnant plasma samples as well as control (j) non-pregnant plasma.
